# Human amnion epithelial cell therapy reduces hypertension-induced vascular stiffening and cognitive impairment

**DOI:** 10.1038/s41598-024-52214-0

**Published:** 2024-01-22

**Authors:** Quynh Nhu Dinh, Cecilia Lo, David Wong Zhang, Vivian Tran, Tayla Gibson-Hughes, Ashleigh Sheriff, Henry Diep, Hyun Ah Kim, Shenpeng R. Zhang, Liz J. Barreto-Arce, Maria Jelinic, Antony Vinh, Thiruma V. Arumugam, Siow Teng Chan, Rebecca Lim, Grant R. Drummond, Christopher G. Sobey, T. Michael De Silva

**Affiliations:** 1 Department of Microbiology, Anatomy, Physiology and Pharmacology, Centre for Cardiovascular Biology and Disease Research, School of Agriculture, Biomedicine and Environment, La Trobe University, Bundoora, VIC 3086 Australia; 2 The Ritchie Centre, Hudson Institute of Medical Research, Clayton, VIC Australia

**Keywords:** Hypertension, Cerebrovascular disorders, Dementia, Inflammation

## Abstract

Vascular inflammation and fibrosis are hallmarks of hypertension and contribute to the development of cardiovascular disease and cognitive impairment. However, current anti-hypertensive drugs do not treat the underlying tissue damage, such as inflammation-associated fibrosis. Human amnion epithelial cells have several properties amenable for treating vascular pathology. This study tested the effect of amnion epithelial cells on vascular pathology and cognitive impairment during hypertension. Male C57Bl6 mice (8–12 weeks) were administered vehicle (saline; n = 58) or angiotensin II (0.7 mg/kg/d, n = 56) subcutaneously for 14 d. After surgery, a subset of mice were injected with 10^6^ amnion epithelial cells intravenously. Angiotensin II infusion increased systolic blood pressure, aortic pulse wave velocity, accumulation of aortic leukocytes, and aortic mRNA expression of collagen subtypes compared to vehicle-infused mice (n = 9–11, *P* < 0.05). Administration of amnion epithelial cells attenuated these effects of angiotensin II (*P* < 0.05). Angiotensin II-induced cognitive impairment was prevented by amnion epithelial cell therapy (n = 7–9, *P* < 0.05). In the brain, amnion epithelial cells modulated some of the inflammatory genes that angiotensin II promoted differential expression of (n = 6, p-adjusted < 0.05). These findings suggest that amnion epithelial cells could be explored as a potential therapy to inhibit vascular pathology and cognitive impairment during hypertension.

## Introduction

Hypertension affects ~ 30% of the global adult population and is the major risk factor for development of cardiovascular disease^1^. While current anti-hypertensive drugs are effective at lowering blood pressure, they typically do not treat the underlying tissue injury, such as inflammation-associated fibrosis.

Excessive fibrosis can lead to vascular stiffening which occurs with ageing and is amplified by hypertension. Vascular stiffening can also precede hypertension^2^ and is itself a major independent risk factor for cardiovascular disease^3^. Aortic stiffening in particular is known to contribute to severe end organ damage, including in the brain where it is associated with increased incidence of cognitive impairment^4^. Therefore, therapies that target vascular fibrosis could reduce the burden of hypertension and cardiovascular disease as well as cognitive decline.

Cell-based therapies could be advantageous over single pharmacological agents, as cells may be able to release multiple mediators which could more effectively target complex disease mechanisms^5^. Amnion epithelial cells are derived from the amnion layer which forms part of the sac that encloses the developing foetus^6^. Amnion epithelial cells have several advantages over other types of cells being considered for therapy, including low immunogenicity, anti-tumourigenic properties, non-invasive extraction procedures and minimal ethical concerns regarding their collection and use^6^. Furthermore, amnion epithelial cells possess anti-fibrotic, anti-inflammatory, anti-apoptotic and pro-angiogenic properties which may be useful in treating cardiovascular diseases^7–9^.

Thus, we tested whether administration of amnion epithelial cells could limit aortic fibrosis and stiffening during experimental hypertension, and whether such an effect is associated with improved cognition.

## Materials and methods

### Animals

This study was approved by the La Trobe University Animal Ethics Committee (AEC19026 and AEC16-93). All procedures were performed in accordance with the ARRIVE guidelines and the National Health and Medical Research Council of Australia code for the care and use of animals for scientific purposes. Male C57BL/6J (WT) mice (n = 114; 8–12 weeks old) were obtained from an in house breeding colony. Standard rodent chow and drinking water were provided ad libitum. Animals were housed with littermates in individually ventilated cages. Mice were randomly assigned to treatment groups and investigators were blinded to treatment groups where possible. As young female mice are known to have a blunted response to angiotensin II infusion^10^, it is not a reliable model to study a potential therapy for hypertension in the female sex. Thus, we only used male mice in this study.

### Administration of angiotensin II and measurement of blood pressure

Mice were infused with saline or angiotensin II (0.7 mg/kg/d S.C.) for 14 days by osmotic minipump (Alzet model 2002) implanted S.C. in the mid-scapular region^11^. Mice were anaesthetised with isoflurane (2–4% inhaled with oxygen [0.4 L/min]) delivered via a heated nosecone and placed on a heat mat. Depth of anaesthesia was monitored by observing respiration rate and checking for absence of a reflex response to a toe pinch. At the time of surgery, mice were administered bupivacaine (2.5 mg/kg S.C.) at the surgical site and carprofen (5 mg/kg S.C.). Mice then received carprofen (5 mg/kg S.C.) daily for 2 days after surgery.

Systolic blood pressure was monitored in conscious mice via tail cuff plethysmography (MC4000 Multichannel system, Hatteras Instruments). Prior to surgery, mice were trained for 1 day (i.e. on day − 1) to acclimatize to the procedure, and blood pressure was then recorded on days 0 (prior to surgery) and 14 post-surgery.

### Preparation and injection of amnion epithelial cells

Amnion epithelial cells were isolated from term placentae donated by healthy volunteers who underwent elective caesarean section delivery as described previously^7^. Saline (vehicle) or 1 × 10^6^ amnion epithelilal cells were injected into the tail vein after the mice regained consciousness from osmotic minipump implantation. In total, 7 mice died within 5 min after bolus injection of cells due to probable pulmonary embolism.

### Assessment of aortic stiffening

Ultrasound imaging of the abdominal aorta was performed on days 0 and 14 post-surgery using a Vevo 2100 (VisualSonics; FUJIFILM; Canada)^12^. Mice were anaesthetised using isoflurane (2% at 0.4 L/min) and placed on a heated platform. Abdominal fur was removed and ultrasound transmission gel (Aquasonics, USA) was applied to the abdomen. Pulse wave doppler images and EKV retrospective acquired B-Mode images were obtained from longitudinal sections of the abdominal aortas (suprarenal) using a MS-400 ultrasound transducer. Data were exported and pulse wave velocity was analysed using the VevoLab and VevoVasc software (FUJIFILM Visualsonics Inc. Canada) and InD-V loop method.

### Flow cytometric analysis of leukocytes in the aorta

Flow cytometry was performed as previously described in brain tissue but modified for the aorta and blood^13^. Mice were killed by carbon dioxide asphyxiation and perfused via the left ventricle with 0.2% clexane (400 IU, Sanofi Aventis, Australia) in 0.01 M phosphate-buffered saline (PBS). The entire aorta (aortic arch to femoral bifurcation) with perivascular fat was harvested for flow cytometry. Samples were minced with scissors and digested in PBS (with MgCl_2_ and CaCl_2_) containing a mixture of collagenase type XI (125 U/ml), collagenase type I-S (460 U/ml) and hyaluronidase (60 U/ml) (Sigma-Aldrich, USA) for 30 min at 37 °C. Samples were then passed through a 70 μm filter.

Whole blood was collected via the right ventricle into heparinised tubes. Samples were then incubated with 10 mL of red blood cell (RBC) lysis buffer for 5 min at room temperature on a shaker. Samples were washed with PBS and centrifuged at 4 °C for 5 min (1500 RPM). This was repeated to ensure that erythrocytes were removed from the sample. Cell were then resuspended with 500 μL PBS.

Cells were stained with an antibody cocktail of anti-CD45 APC-Cy7 (30-F11, Biolegend, USA), anti-CD11b Pacific Blue (M1-70, eBioscience, USA), anti-Ly6G PE-Cy7 (1A8, Biolegend, USA), anti-Ly6C FITC (HK1.4, Biolegend, USA), anti-CD3ε APC (145-2C11, eBioscience, USA), anti-CD4 Alexa Fluor 700 (GK1.5, eBioscience, USA), anti-F4/80 BV711 (BM8, Biolegend, USA) and anti-CD19 PE-Cy5 (6D5, Biolegend, USA), diluted in PBS containing 0.5% bovine serum albumin. Samples were analysed via flow cytometry using a CytoFLEX LX flow cytometer (Beckman Coulter, USA) and FlowJo Software (version 10.1, Tree Star Inc, USA, gating strategy shown in Supplementary Fig. 1). Cell numbers were expressed as total cells per aorta.

### Gene expression in the aorta

Messenger RNA expression of collagen in the aorta was determined using TaqMan^®^ real-time PCR. The aorta was harvested and snap frozen in liquid nitrogen. Aortae were sonicated in TRIzol™ (Life Technologies, USA), mixed with chloroform, and centrifuged at 824 × *g* for 15 min at 4 °C. The aqueous phase was collected and RNA was extracted using the RNeasy^®^ Micro Kit (Qiagen, USA). RNA was quantified using a NanoDrop One spectrophotometer (Thermo Scientific, USA) and converted to 1^st^ strand cDNA using High Capacity cDNA RT Kit (Applied Biosystems, USA). Commercially available primers (Applied Biosystems, USA) were used to measure mRNA expression of collagen (*Col1a1*, *Col3a1*, *Col4a1*, *Col5a1*), and a house-keeping gene, *Gapdh*, on a CFX96 Touch Real-Time PCR Detection machine (Bio-Rad, USA). Changes in gene expression were assessed using the delta-delta C_T_ method^14^.

### Immunolocalisation of amnion epithelial cells

Amnion epithelial cells were localised in the aorta using immunohistochemistry. Fixed (4% paraformaldehyde), paraffin-embedded thoracic aorta sections (5 μm) were dewaxed, incubated with histolene (2 × 10 min), rinsed with 100% and 70% ethanol and then distilled H_2_O. Antigen retrieval was performed using citrate buffer (pH 6.0), sections were then washed with PBS and endogenous peroxidase was blocked with 1% H_2_O_2_. Endogenous mouse IgG was blocked using goat anti-mouse IgG followed by blocking with 10% donkey serum in phosphate buffered saline. Sections were then incubated overnight at 4 °C with anti HLA-G (1:500; Ab52455; Abcam, UK). The next day, sections were washed and incubated with a horse radish peroxidase-conjugated donkey anti-mouse secondary antibody (1:200) for 45 min at room temperature. Sections were washed with PBS and incubated in DAB brown solution. Following this, sections were washed, counterstained with haemotoxylin and mounted with DPX mounting media. Images were captured with an Olympus DP73 Camera (Olympus Corporation, Tokyo, Japan) connected to an Olympus BX53 microscope (Olympus Corporation, Tokyo, Japan) at 400 × magnification running CellSens Standard Software (version 1.17, Olympus Corporation).

### Picrosirius red staining

For picrosirius red staining, aortae were prepared and sectioned as described above. Five μm sections were incubated with 0.3% Picrosirius red solution (PolysciencesInc.,USA) for 1.5 h at room temperature. Sections were washed with acidified water, rinsed with 100% ethanol and histolene before moutning with DPX mounting media. Sections were imaged using a polarized microscope (Olympus BX53, Japan) and analysed for percentage collagen content by ImageJ. Two aortic sections per mouse were analysed by an investigator blinded to the treatment groups.

### Behavioural testing

We used the open field test to evaluate locomotor activity and anxiety-like behavior^15,16^, and working memory using the novel object recognition test^17^. Mice were acclimatized to the testing apparatus by placing them in the empty box for 10 min per day for 2 days prior to testing. The open field test was performed on day 2 of acclimation. A 30 × 30 cm zone was set up in the middle of the box (inner zone) and a 10 cm wide zone around the edges of the box (outer zone). Time in the inner zone as well as total distance travelled was tracked using Ethovision XT (Noldus Information Technology BV, Wageningen, The Netherlands). On the day of testing, the mouse was placed in the box containing two identical objects and allowed to explore for 10 min. One hour later, the mouse was placed back in the same box for 5 min with one of the familiar objects replaced by a novel object. Interactions with the objects (defined as nose entering a 2 cm zone around the object) were tracked using Ethovision XT. Mice than did not interact with the objects for > 10 s in total were excluded from the analysis.

### Brain RNA sequencing

RNA sequencing was performed as previously described^18^. In brief, brain hemispheres were harvested and snap frozen in liquid nitrogen. Brains were sonicated in TRIzol™ (Life Technologies, USA), mixed with chloroform, and centrifuged at 824 × *g* for 15 min at 4 °C. The aqueous phase was collected and RNA was extracted using the RNeasy^®^ Micro Kit (Qiagen, USA). RNA was quantified using a NanoDrop One spectrophotometer (Thermo Scientific, USA) and then stored at − 80 °C. The RNA samples were sent to NovogeneAIT Genomics (Singapore) for cDNA library preparation and RNA sequencing. mRNA was purified from total RNA using poly-T oligo-attached magnetics. mRNA was converted to cDNA and purified using AMPure XP Beads (Beckman Coulter Life Sciences, USA). cDNA libraries were acquired by PCR amplification. High-throughput sequencing was conducted using the HiSeqTM2500 platform (Illumina, USA). The results were mapped to the Ensembl-released mouse genome sequence and annotation. Differential expression analysis was conducted using the DESeq R Package V.1.10.1 and *P*-values were adjusted using the Banjamini and Hochberg’s approach for controlling the false discovery rate. Genes were considered differentially expressed if the adjusted *P*-value was less than 0.05. R package heatmap3 and log2Fold-Change output from EdgeR V.3.2.4 were used to create heatmaps for differentially expressed genes.

### Statistical analysis

Results are expressed as mean ± S.E.M. Sample size calculations were performed with G Power Software (version 3.1.5). Normality of the data was checked using a Shapiro–Wilk test and was normally distributed. Statistical analyses between groups were performed using Student’s unpaired t-test, one-sample t-test, or a one- or two-way ANOVA followed by a Tukey’s or Sidak’s post-hoc test, as appropriate. *P* < 0.05 was considered to be significant. RNA sequencing data were analysed using R Studio. GraphPad Prism software (version 9.0, GraphPad Software Inc., USA) was used to perform all other statistical analyses.

## Results

### Amnion epithelial cells infiltrate the aorta in hypertensive mice

**Figure 1:**
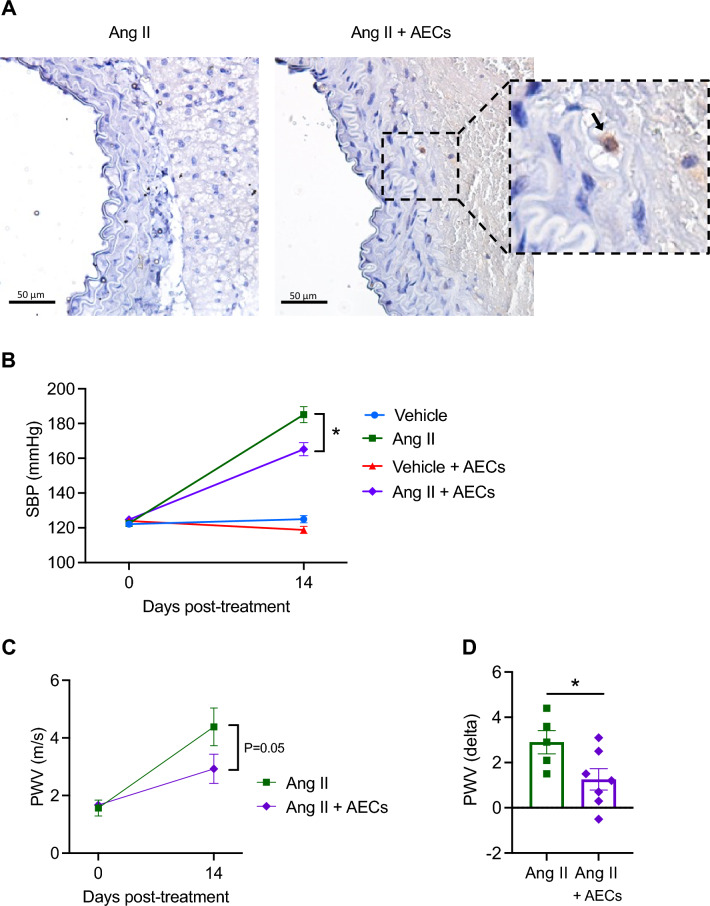
Angiotensin II induced-hypertension and aortic stiffening is reduced by administration of amnion epithelial cells. (**A**) Immunostaining for HLA-G in angiotensin II and angiotensin II + amnion epithelial cell (AEC) treated mice. Nuclei were stained with haemotoxylin. 400X magnification, scale bar = 50 μm. Representative of n = 3. (**B**) Effect of amnion epithelial cells (AECs) on angiotensin II-induced hypertension (n = 8–11). All data are mean ± S.E.M. *P < 0.05. Two-way ANOVA with Tukey’s test. (**C**) Effect of angiotensin II infusion and co-treatment with AECs on pulse wave velocity (n = 6–7). All data are mean ± S.E.M. *P < 0.05. Two-way ANOVA with Sidak’s test. (**D**) Delta change (day 0 and 14) of pulse wave velocity (n = 6–7). All data are mean ± S.E.M. *P < 0.05. Student’s unpaired t-test.

To localise amnion epithelial cells in the aorta, we stained sections with human leukocyte antigen G (HLA-G)^19^. As expected, no HLA-G positive staining was observed in angiotensin II-infused mice that received saline injection (Fig. 1A). By contrast, HLA-G positive cells (arrow in higher magnification view) were observed in the adventitia of angiotensin II-infused mice that had received amnion epithelial cells 14 d previously (Fig. 1A). No positive staining was observed in medial or intimal layers of the aorta.

### Amnion epithelial cells reduce systolic blood pressure and aortic pulse wave velocity

Infusion of angiotensin II for two weeks resulted in an elevation in systolic blood pressure of ~ 60 mmHg (Fig. 1B). Administration of amnion epithelial cells blunted the pressor response to angiotensin II by 20 mmHg (Fig. 1B). Treatment with amnion epithelial cells did not affect systolic blood pressure in vehicle-infused mice (Fig. 1B). Aortic pulse wave velocity measured by ultrasound sonography was lower in angiotensin II-infused mice treated with amnion epithelial cells (Fig. 1C,D).

### Amnion epithelial cells reduce infiltration of immune cells into the aorta

**Figure 2:**
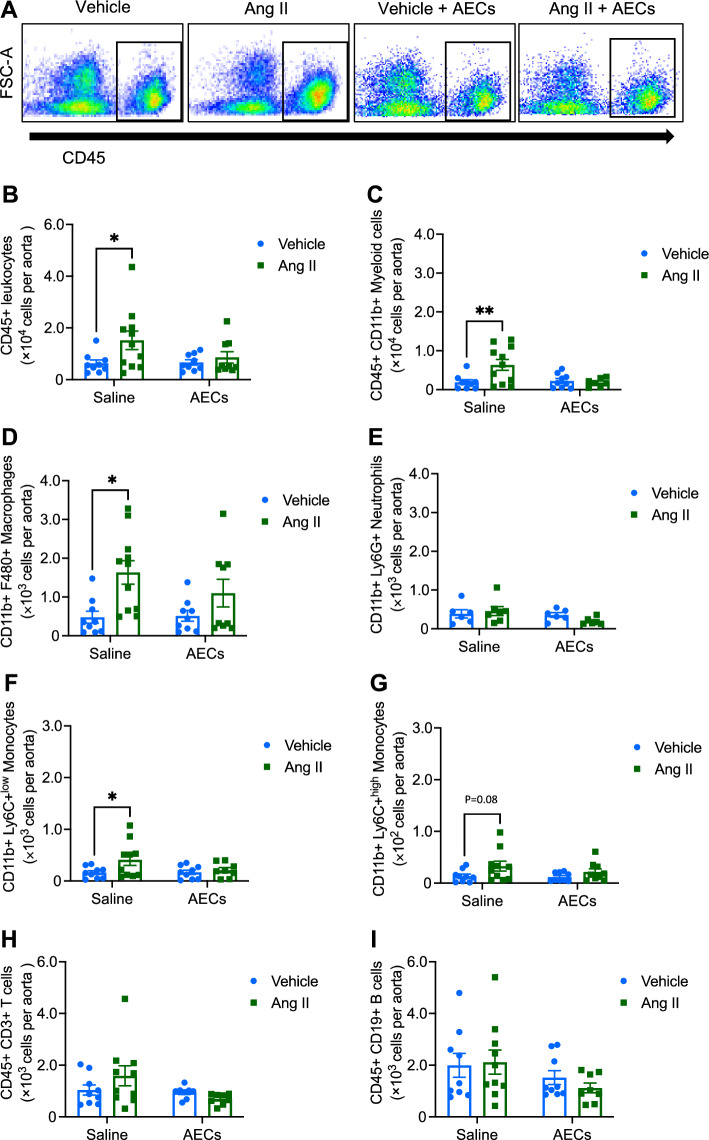
Administration of amnion epithelial cells prevents angiotensin II-induced aortic immune cell infiltration. (**A**) Representative flow cytometry dot plots showing gating strategy for total leukocytes (CD45+ high) from the aorta of mice infused with vehicle, angiotensin II, vehicle + amnion epithelial cells (AECs) and angiotensin II + AECs. The effect of angiotensin II infusion and co-treatment with AECs on (**B**) CD45+ leukocytes, (**C**) CD11b+ myeloid cells, (**D**) F4/80+ macrophages, (**E**) Ly6G+ neutrophils, (**F**) Ly6C+ low monocytes, (**G**) Ly6C+ high monocytes, (**H**) CD3+ T cells and (**I**) CD19+ B cells in the aorta (n = 8–11). All data are mean ± S.E.M. *P < 0.05. Two-way ANOVA with Sidak’s test.

Angiotensin II infusion increased the number of total leukocytes (CD45+; Fig. 2A,B), myeloid cells (CD11b+; Fig. 2C), macrophages (F4/80+; Fig. 2D) and Ly6C^low^ monocytes (Ly6C+; Fig. 2F) in the aorta. Numbers of aortic neutrophils (Ly6G+; Fig. 2E), Ly6C^high^ monocytes (Ly6C+; Fig. 2G), T cells (CD3+; Fig. 2H) and B cells (CD19+; Fig. 2I) were not altered by angiotensin II infusion. Administration of amnion epithelial cells prevented the angiotensin II-induced increases in total leukocytes (Fig. 2A-B), myeloid cells (Fig. 2C), macrophages (Fig. 2D) and Ly6C^low^ monocytes (Fig. 2F) in the aorta.

Additionally, we quantified circulating leukocytes. We did not observe any change in CD45+ leukocytes in hypertensive mice treated with amnion epithelial cells (angiotensin II: 1.1 ± 0.4 × 10^6^, vs angiotensin II + amnion epithelial cells: 1.1 ± 0.7 × 10^6^, cells per mL of blood. n = 4–5 per group).

### Amnion epithelial cells reduce aortic expression of collagen genes

**Figure 3:**
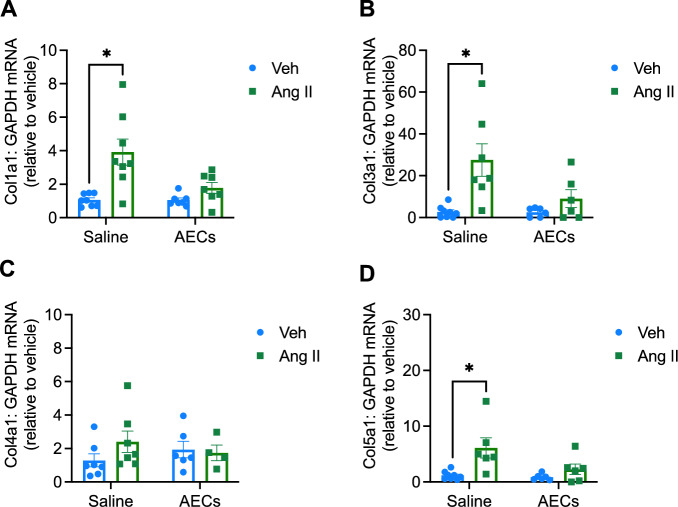
Administration of amnion epithelial cells prevents angiotensin II-induced aortic expression of collagen. The effect of angiotensin infusion and co-treatment with amnion epithelial cells (AECs) on mRNA expression of (**A**) collagen type 1 alpha 1 chain, (**B**) collagen type 3 alpha 1 chain, (**C**) collagen type 4 alpha 1 chain and (**D**) collagen type 5 alpha 1 chain in the aorta (n = 6–8). All data are mean ± S.E.M. *P < 0.05. Two-way ANOVA with Sidak’s test.

Angiotensin II infusion increased mRNA expression of *Col1a1*, *Col3a1* and *Col5a1* (Fig. 3A, B and D) but not *Col4a1* (Fig. 3C). These changes in gene expression were prevented by administration of amnion epithelial cells (Fig. 3A, B and D). Aortic collagen deposition was also assessed using Picrosirius red staining (Supplementary Fig. 2). We did not observe any significant changes in collagen 1, collagen 3 or the collagen 1:collagen 3 ratio.

### Amnion epithelial cells improve working memory in hypertensive mice

**Figure 4:**
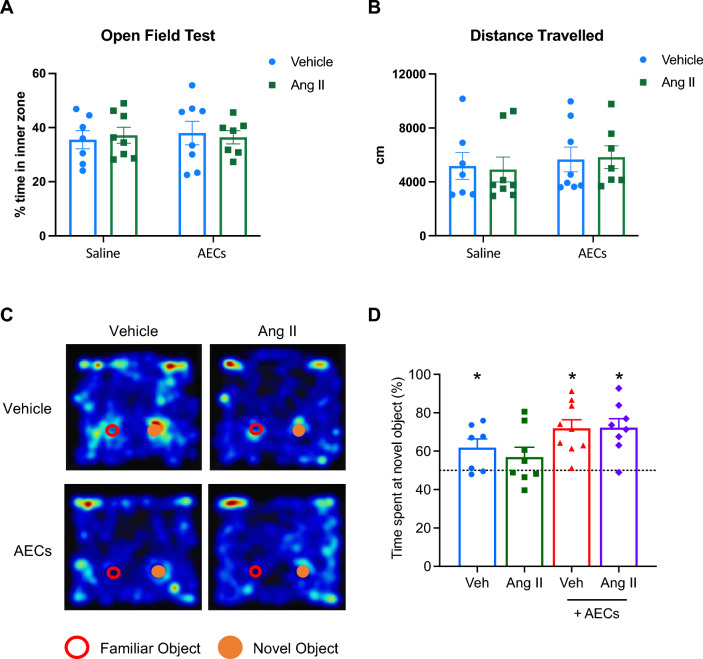
Administration of amnion epithelial cells improves angiotensin II-induced cognitive impairment. (**A**) Percentage of time spent in the inner zone in the open field test. (**B**) Total distance travelled (in cm) in the open field test (**C**) Representative heatmap plots showing interaction between familiar and novel objects in mice infused with vehicle, angiotensin II, vehicle + amnion epithelilal cells (AECs) and angiotensin II + AECs. (**D**) Effect of angiotensin II infusion and co-treatment of AECs on recognition memory (n = 7–9). All data are mean ± S.E.M. *P < 0.05. One-sample t-test versus 50%.

Neither the % time spent in the inner zone (Fig. 4A) or total distance travelled (Fig. 4B) was affected by hypertension or amnion epithelial cell administration. Intact working memory was defined as spending significantly more than 50% of the interaction time exploring the novel object. Representative heat maps for each treatment group with the location of novel and familiar objects are shown in Fig. 4C. Vehicle-infused mice spent more time (~ 60%) interacting with the novel object than the familiar object (Fig. 4D; one sample t-test vs 50%), whereas angiotensin II-infused mice did not discriminate between novel and familiar objects (Fig. 4D). Amnion epithelial cell therapy did not adversely impact the performance of vehicle-infused mice (Fig. 4D). Moreover, administration of amnion epithelial cells in angiotensin II-infused mice resulted in more time spent interacting with the novel object than the familiar object (~ 70%; Fig. 4B).

### Amnion epithelial cells modulate expression of genes in the brain

**Figure 5:**
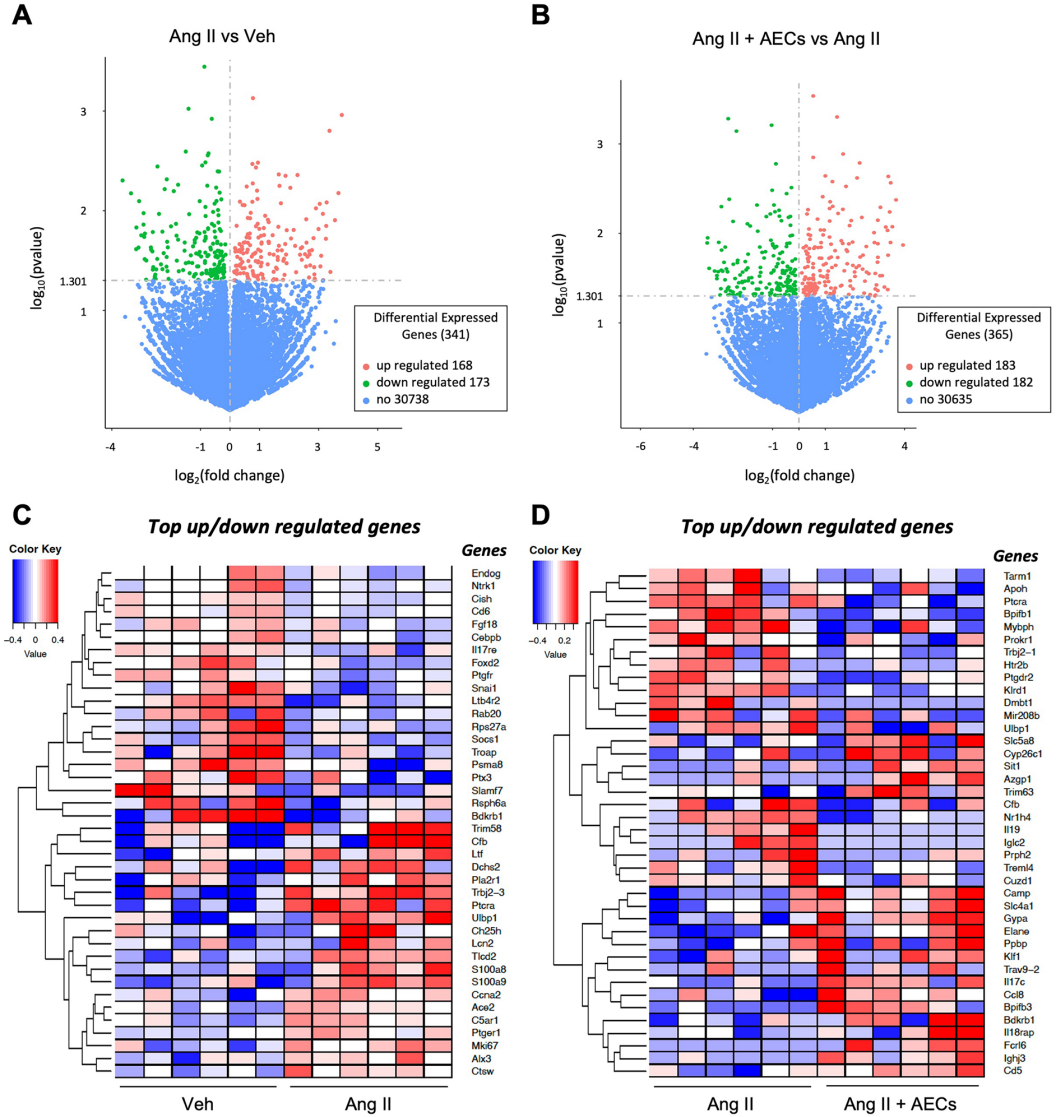
Administration of amnion epithelial cells modulates genes that are differentially expressed in the brain by angiotensin II-infusion. Volcano plot of differentially expressed genes in brains of mice infused with (**A**) vehicle versus angiotensin II or (**B**) angiotensin II versus angiotensin II + AECs. The threshold of differential expression is *p*-value < 0.05. The horizontal axis is the log2 fold change of genes. The vertical axis is statistical significance scaled as − log 10 *p*-value. Each dot represents an individual gene (blue: no significant difference; red: upregulated expression; green: downregulated expression). The top upregulated and downregulated genes in brains of mice infused with (**C**) vehicle versus angiotensin II or (**D**) angiotensin II versus angiotensin II + AECs (n = 6 per group). Upregulated genes in red and downregulated genes in blue. The colour scale represents the log10 (average FPKM + 1) value.

Bulk RNA sequencing was performed on the brains of mice infused with vehicle, angiotensin II, vehicle + amnion epithelial cells or angiotensin II + amnion epithelial cells. A heatmap showing all differentially expressed genes is shown in Supplementary Fig. 3. When comparing mice infused with angiotensin II alone versus vehicle alone, there were 341 differentially expressed genes (168 upregulated and 173 downregulated) in the brain (Fig. 5A). There were 365 differentially expressed genes (183 upregulated and 182 downregulated) in the brain when comparing mice infused with angiotensin II alone versus angiotensin II + amnion epithelial cells (Fig. 5B).

Further analysis of genes related to inflammation revealed that the genes most downregulated by angiotensin II compared to vehicle included endonuclease G (*Endog*), neurotrophic tyrosine kinase receptor (*Ntrk1*), cytokine inducible SH2 containing protein (*Cish*), CD6 antigen (*Cd6*), CCAAT enhancer binding protein beta (*Cebpb*), interleukin 17 receptor E (*Il17re*), leukotriene B4 receptor 2 (*Ltb4r2*), suppressor of cytokine signalling 1 (*Socs1*), pentrasin 3(*Ptx3*) and SLAM family member 7 (*Slamf7*) (Fig. 5C). The top upregulated genes following infusion with angiotensin II included complement factor B (*Cfb*), lactotransferrin (*Ltf*), dachsous cadherin-related 2 (*Dchs2*), T cell receptor beta joining 2-3 (*Trbj2-3*), pre T cell antigen receptor alpha (*Ptcra*), UL16 binding protein 1 (*Ulbp1*), cholesterol 25-hydroxylase (*Ch25h*), lipocalin 2 (*Lcn2*), S100 calcium binding protein A8 (*S100a8*), S100 calcium binding protein A9 (*S100a9*), angiotensin-converting enzyme 2 (*Ace2*), complement C5a receptor 1 (*C5ar1*) and cathepsin W (*Ctsw*) (Fig. 5C).

Compared to angiotensin II + saline treatment, the top downregulated genes in brains from angiotensin II + amnion epithelial cell treated mice include T cell-interacting, activating receptor on myeloid cells 1 (*Tarm1*), apolipoprotein H (*Apoh*), pre T cell antigen receptor alpha (*Ptcra*), B lymphocyte kinase (*Blk*), BPI fold containing family member B member (*Bpifb1*), prokineticin receptor 1 (*prokr1*), T cell receptor beta joining 2-1 (*Trbj2-1*), prostaglandin D2 receptor 2 (*Ptgdr2*), deleted in malignant brain tumours 1 (*Dmbt1*), UL16 binding protein 1 (*Ulbp1*), complement factor B (*Cfb*) and triggering receptor expressed on myeloid cells-like protein (*Treml4*) (Fig. 5D). The top upregulated genes in brains from mice treated with angiotensin II + amnion epithelial cells include alpha-2-glycoprotein 1, zinc-binding (*Azgp1*), cathelicidin antimicrobial peptide (*Camp*), pro-platelet basic protein (*Ppbp*), interleukin 17c (*Il17c*), C-C motif chemokine ligand 8 (*Ccl8*), BPI fold containing family member B member interleukin 18 receptor accessory protein (*Il18rap*), Fc receptor like 6 (*Fcrl6*) and T cell surface glycoprotein CD5 (Cd5) (Fig. 5D).

## Discussion

The major finding of this study is that amnion epithelial cells can prevent aortic stiffening, inflammation and cognitive impairment induced by hypertension. Specifically, amnion epithelial cells limited the pressor response to angiotensin II, prevented angiotensin II-induced aortic immune cell infiltration and expression of collagen, prevented impairment of working memory and modulated angiotensin II-induced transcriptomic changes in the brain. Our findings are consistent with some of the known anti-inflammatory and anti-fibrotic properties of amnion epithelial cells. Thus, amnion epithelial cells may be a potential therapeutic option for the prevention or treatment of hypertension-induced vascular injury and cognitive impairment.

Amnion epithelial cells blunted the pressor response to angiotensin II by 20 mmHg. Previously, amnion epithelial cells have been found to prevent pulmonary hypertension in experimental lung injury^20^. To our knowledge, this study is the first to examine the effects of amnion epithelial cells in a model of systemic hypertension. The blood pressure lowering effect of amnion epithelial cells could at least partly be due to their immunomodulatory properties. There is now a large body of evidence supporting a role of the immune system in hypertension^21^, with immune cells such as T cells^22^ and macrophages^23^ having been demonstrated to contribute to hypertension. In this study, angiotensin II promoted infiltration of leukocytes (specifically macrophages and inflammatory monocytes) into the aorta, and this could be prevented by co-adminstration of amnion epithelial cells. We have previously shown that amnion epithelial cells home to sites of acute injury (i.e. stroke^7^ or traumatic brain injury^19^). In the present study we identified amnion epithelial cells in the aorta, which we and others have shown to be a site of inflammation and injury during hypertension. As hypertension causes systemic injury, it is also possible that amnion epithelial cells migrate to other tissues. We have also shown that a CCR2 antagonist reduces blood pressure and aortic macrophage numbers in mice infused with angiotensin II^24^. Similarly, depletion of monocytes and macrophages via treatment with clodronate^23^ or low-dose diptheria toxin^25^ attenuated the pressor response to angiotensin II and this was restored by adoptive transfer of monocytes^25^. Furthermore, we have reported these cells to have anti-inflammatory properties in animal models of lung injury^26,27^ and stroke^7^. In this study, amnion epithelial cells reduced macrophage and monocyte infiltration in the aorta. Amnion epithelial cells can inhibit macrophage migration in vitro through production of macrophage migration-inhibitory factor (MIF)^28^. We previously demonstrated that amnion epithelial cells can reduce macrophage infiltration in a mouse model of lung injury, and decrease chemotaxis of macrophages towards recombinant mouse macrophage inflammatory protein 2 in vitro^29^. Hence, it is possible that amnion epithelial cells in this study reduced aortic macrophage infiltration at least in part by inhibiting their migration. Amnion epithelial cells do not appear to affect macrophage proliferation or survival^28,29^. Thus, the anti-hypertensive effect of amnion epithelial cells may, in part, be due to their ability to suppress innate immune cell infiltration into the aorta.

We and others have shown that amnion epithelial cells can reduce fibrosis in mouse models of liver fibrosis^8,30,31^ and hepatic stellate cells co-cultured with amnion epithelial cells have reduced collagen production^8,32^. Vascular stiffening results from structural changes such as excessive fibrosis (including increased collagen) and reduced elastin. Angiotensin II infusion increased both expression of aortic collagen genes and aortic pulse wave velocity in vivo^33^, consistent with the development of aortic stiffening^12^. By contrast, administration of amnion epithelial cells reduced expression of collagen and pulse wave velocity in angiotensin II-infused mice, consistent with anti-fibrotic actions that limited vascular stiffness in hypertension.

Angiotensin II caused cognitive impairment (impaired working memory assessed using the novel object recognition test), but did not impair locomotor activity or anxiety-like behaviour, and this was prevented by amnion epithelial cells. This protection of working memory could have occurred at least in part by reducing vascular stiffening. Vascular stiffening is a predictor of cognitive decline^34^ and increases in aortic pulse wave velocity are associated with impaired memory and executive function^4,35^. Increased pulse wave velocity reflects elevated pulsatile pressure in the brain which can damage the cerebral vasculature^36^ and lead to breakdown of the blood brain barrier (BBB), neuroinflammation, neurodegeneration and cognitive decline^37^. It is also possible that the BBB breakdown that occurs with hypertension^38^ may enable amnion epithelial cells to enter the brain parenchyma and protect against hypertension-induced cognitive impairment. Other anti-fibrotic therapies have also been reported to improve cognition. Interestingly, idiopathic pulmonary fibrosis is associated with aortic stiffening^39^ and cognitive deficits^40^. Furthermore, pirfenidone, an anti-fibrotic and anti-inflammatory drug used to treat idiopathic pulmonary fibrosis, can prevent scopolamine-induced cognitive impairment^41^, improve neurological outcome in a rat model of traumatic brain injury^42^, and inhibit angiotensin II-induced cardiac hypertrophy and fibrosis^43^. A limitation of the novel object recognition test is that it cannot distinguish between degrees of impairment. Thus, we can only conclude that angiotensin II treatment impaired working memory and that amnion epithelial cells prevented this. To determine degrees of impairment, a different cognitive test (e.g. Barnes maze) would need to be performed.

Perivascular macrophages have been shown to contribute to hypertension-induced cognitive impairment^44^. The effect of amnion epithelial cells on perivascular macrophages is not currently known, however, amnion epithelial cells have been reported to reduce microglial activation in the brain in a rat model of stroke^45^. Microglia are the resident macrophages of the brain and microglia activation can contribute to cognitive decline in a mouse model of Parkinson’s disease^46^. Whether amnion epithelial cells modulate the function of perivascular macrophages in hypertension is worthy of future study.

Infusion of angiotensin II caused upregulation of genes in the brain involved in biological processes such as inflammation and immunity (*Cfb, Dchs2*, *Trbj2-3, Ptcra, Ulbp1, Lcn2, S100a8, S100a9, C5ar1, Ctsw*), lipid metabolism (*Ch25h*) and production of angiotensin 1-7 (*Ace2*). Infusion of angiotensin II caused downregulation of genes in the brain involved in survival of neurons (*Ntrk1*), neuronal repair and regeneration (*Ptx3*) and anti-inflammatory pathways (*Cish, Socs1*). Angiotenisn II is known to have pro-inflammatory^47^ and pro-apoptotic^48^ effects in the brain which can contribute to cognitive decline. Some of the genes that angiotensin II promoted differential expression of, such as *Dchs2*^49^*, Ch25h*^50^*, S100a8*^51^*, S100a9*^52^*, Ntrk1*^53^ and *Ptx3*^54^*,* are associated with the most common form of dementia, Alzheimer’s disease. Thus, we speculate that that these genes may have contributed to the cognitive impairment observed in hypertension. Furthermore, our data show that angiotensin II upregulated expression of pro-inflammatory genes *Cfb, Ptcra* and *Ulbp1* in the brain, and their expression was downregulated by combined administration of angiotensin II and amnion epithelial cells. Complement factor B (*Cfb*) is a component of the alternative complement pathway, the inhibition of which improves cognition in mouse models of stroke^55^ and traumatic brain injury^56^. *Ptcra* forms the alpha chain of the pre-T cell receptor which regulates T cell development^57^. Previously, we have shown that the angiotensin II model of hypertension increases T cell infiltration in the brain^58^ and T cells have been reported to promote cognitive impairment in a mouse model of Alzheimer’s disease^59^ and taupathy^60^. *Ulbp1* is a ligand for the NKG2D receptor found on natural killer cells and depletion of natural killer cells improves cognition in mouse models of ageing^54^ and Alzheimer’s disease^61^. As the alternative complement pathway, T cells and natural killer cells can contribute to the development of cognitive impairment, amnion epithelial cells may have preserved cognition by modulating these inflammatory pathways. Furthermore infusion with angiotensin II + amnion epithelial cells also downregulated other genes involved in apoptosis (*Treml4*), macrophage (*Tarm1*), B cell (*Blk*) and T cell (*Trbj2-1*) immunity which may have also contributed to the protective effects of the amnion epithelial cells on cognition. Thus, differential expression of several genes in the brain during hypertension that are modulated by amnion epithelial cell therapy are likely to have contributed to cognitive function in these studies.

Overall, the present study has demonstrated that amnion epithelial cells can reduce systolic blood pressure, vascular stiffening, inflammation and cognitive impairment in angiotensin II-infused mice. In particular, we suggest that the protective effect of amnion epithelial cells against the development of aortic stiffening may have been an important contributing factor to the preservation of cognition during hypertension.

### Supplementary Information

Supplementary Figures.

## Data Availability

The data that support the findings of this study are available from the corresponding author upon reasonable request. The datasets for bulk RNA sequencing discussed in this publication have been deposited in NCBI's Gene Expression Omnibus^62^ and are accessible through GEO Series accession number GSE248059 (https://www.ncbi.nlm.nih.gov/geo/query/acc.cgi?acc=GSE248059).
