# Supplementary material for: Human amnion epithelial cell therapy reduces hypertension-induced vascular stiffening and cognitive impairment
Source: Sci Rep. 2024 Jan 22;14:1837. doi: 10.1038/s41598-024-52214-0 (PMC10800338; doi:10.1038/s41598-024-52214-0)
Supplement: Supplementary file 1 — Supplementary Figures. [file 41598_2024_52214_MOESM1_ESM.docx]

**SUPPLEMENTARY MATERIAL**

**Human Amnion Epithelial Cell Therapy Reduces Hypertension-induced Vascular Stiffening and Cognitive Impairment**

Quynh Nhu Dinh^1^, Cecilia Lo^1^, David Wong Zhang^1^, Vivian Tran^1^, Tayla Gibson-Hughes^1^, Ashleigh Sheriff^1^, Henry Diep^1^, Hyun Ah Kim^1^, Shenpeng R Zhang^1^, Liz J Barreto-Arce^1^, Antony Vinh^1^, Thiruma V Arumugam^1^, Siow Teng Chan^2^, Rebecca Lim^2^, Grant R Drummond^1^, *Christopher G Sobey^1^ & *T. Michael De Silva^1^

^1^Centre for Cardiovascular Biology and Disease Research, Department of Microbiology, Anatomy, Physiology and Pharmacology, La Trobe University, Bundoora, VIC, Australia

^2^The Ritchie Centre, Hudson Institute of Medical Research, Clayton, VIC, Australia

*Corresponding Authors:

T. Michael De Silva, PhD Christopher G Sobey, PhD

Department of Microbiology, Anatomy, Department of Microbiology, Anatomy, Physiology & Pharmacology Physiology & Pharmacology
School of Agriculture, Biomedicine & School of Agriculture, Biomedicine & Environment Environment

La Trobe University La Trobe University

Bundoora, Victoria 3086, Australia Bundoora, Victoria 3086, Australia

e-Mail: [t.desilva@latrobe.edu.au](mailto:t.desilva@latrobe.edu.au) e-Mail: [c.sobey@latrobe.edu.au](mailto:c.sobey@latrobe.edu.au)

Phone: +61-3-94796876 Phone: +61-3-9479 1316

**SUPPLEMENTARY FIGURES**

**
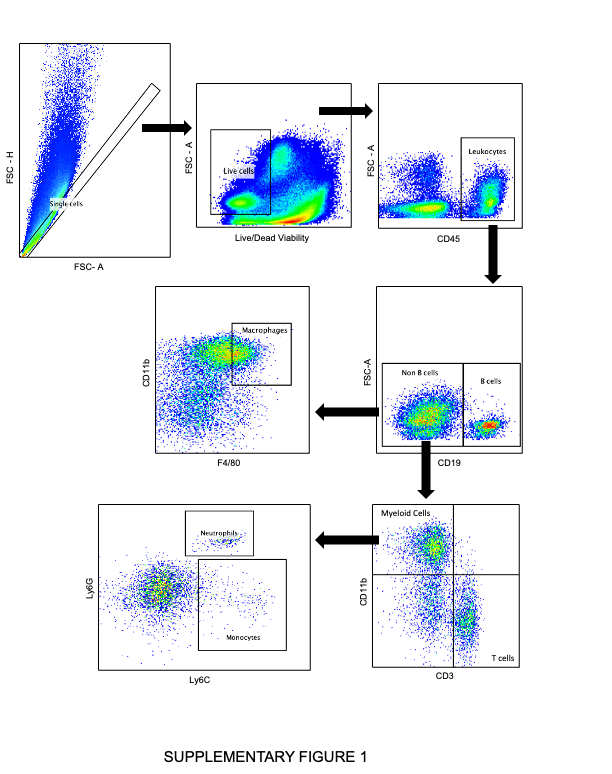
**

**Supplementary Figure 1**

**Gating strategy for flow cytometric analysis.** Single cells were gated by forward scatter (FSC)-height vs FSC-area and dead cells were excluded (live/dead stain). From this, leukocytes were gated as the CD45+ population against FSC-area. Leukocytes were then further gated into B cells (CD45+CD19+) and non B cells (CD45+CD19-). Non B cells were separated into myeloid cells (CD45+CD11b+) and T cells (CD45+CD3+). The myeloid cells were separated as neutrophils (CD45+CD11b+Ly6G+), monocytes (CD45+CD11b+Ly6C+) and macrophages (CD45+CD11b+F4/80+).

**Supplementary Figure 2.** Collagen deposition was assessed by picrosirius red staining and imaged using polarised light. Quantified data showing the percentage area of collagen deposition in the aorta following treatment with angiotensin II ± co-treatment with amnion epithelial cells (AECs). **A:** collagen 1, **B:** collagen 3 and **C:** collagen 1 to collagen 3 ratio in the aorta. Quantification of each collagen 1 and 3 was performed using ImageJ, n=4-7 per group.

**Supplementary Figure 2**

**Heatmap of all genes analysed.** Heatmap of all genes analysed in brain of mice infused with vehicle, angiotensin II, vehicle + amnion epithelilal cells (AECs) and angiotensin II + AECs. Upregulated genes in red and downregulated genes in green.
